# Campbell title registrations to date – August 2024, and discontinued protocols

**DOI:** 10.1002/cl2.70015

**Published:** 2024-12-10

Details of new titles for systematic reviews or evidence and gap maps that have been accepted by the Editor of a Campbell Coordinating Group are published in each issue of the journal. If you would like to receive a copy of the approved title registration form, please *send an email* to the Managing Editor of the relevant Coordinating Group.

A list of discontinued protocols appears below these new titles. If you are interested to continue a project, please get in touch with the Managing Editor of the relevant Coordinating Group or email info@campbellcollaboration.org.

## AGEING

Long distance walking and well‐being: A scoping review

Hind Sabri, Elizabeth Ghogomu, Victoria Barbeau, Tracey Howe, Howard White, Yanfei Li, Qian Liu, Weize Kong, Nina Cruz, Vivian Welch

6 November 2024

Effects of long‐term care interventions for older adults: An evidence and gap map

Elizabeth Tanjong‐Ghogomu, Nadisha Ratnasekera, Paul Hebert, Vivian Welch

30 October 2024

## BUSINESS AND MANAGEMENT

Data pricing: An evidence and gap map

Yufeng Wen, Yajie Zhou, Liping Guo, Wenjie Zhou, Yueyan Zhao, Weijie Lin,

4 November 2024

## CHILDREN AND YOUNG PERSONS WELLBEING

What are the reasons mothers choose human milk as their method of infant nutrition? A mixed methods systematic review

Niamh Ryan, Patricia Leahy‐Warren, Siobhain O'Mahony, Helen Mulcahy, Lloyd Philpott

9 October 2024

Determinants of student well‐being and perceived social support in higher education: A systematic literature review

Dawei Li, Farhana Kamarul Bahrin

23 September 2024

The interventions for Youth Employment in China: A country evidence map

Xiya Shao, Liping Guo, Wenjie Zhou, Yufeng Wen, Weijie Lin, Yueyan Zhao, Yang Yang, Hongli Shang, Yajie Zhou, Yijie Zhang

23 September 2024

## CLIMATE SOLUTIONS

The impact of relocation processes on people facing socio‐territorial iniquities: A scoping review

Pascale Chagnon, Pierre Paul Audate, Geneviève Cloutier

23 August 2024

## CRIME AND JUSTICE

Understanding the structure, content, and impact of far‐right extremist propaganda disseminated online: A systematic review

Mia Doolan, Kiran Sarma

25 October 2024

## DISABILITY

Strategies to enhance inclusion in informed consent practice for people with sensory impairment: A systematic literature review

Fleur O'Hare, Sujani Thrimawithana, Aimee Clague, Camille Paynter, David Foran, Caroline Ondracek, Eden Robertson, Tessa Saunders, Lauren Ayton

24 October 2024

## INTERNATIONAL DEVELOPMENT

Evidence and gap map of climate change adaptation interventions for enhancing food security and livelihoods in sub‐Saharan Africa

David Sarfo Ameyaw, Kwadwo Danso‐Mensah, Joseph Clottey, Clarice Panyin Nyan, Sheila Agyemang Oppong, Miriam Oppong, Nana Esi Badu‐Ansah, Desmond Kaledzi, Isaac Letsa

29 August 2024

Randomized controlled trials for poverty alleviation and environmental conservation in low‐ and middle‐income countries: A systematic review

Adriana Molina Garzon, Aemro Worku, Andres Ramasco, Lakshmi Iyer, Ellis Adams, Krister Andersson, Daniel Miller

29 August 2024

## KNOWLEDGE TRANSLATION AND IMPLEMENTATION

Evaluation of the effectiveness of herbal and natural solutions for disinfection of Gutta Percha before obturation—Systematic Review

Hemalatha Hiremath, Priyankar Roy, Sadanand Kulkarni

22 November 2024

The accuracy and concordance of genotyping platforms for Pharmacogenomic testing: A systematic review protocol of diagnostic test accuracy

Kiflu Tesfamicael, Mike Musker, David Adelson, Martin Lewis

31 October 2024

Value of rural provider‐to‐provider telehealth for emergency and inpatient care: An evidence and gap map

Kaylie Toll, Danika Jurat, Suzanne Robinson, Stephen Andrew, Aled Williams, Zaheerah Haywood, Amy Cole, Joanna Moullin

31 October 2024

The uptake of telemedicine among healthcare professionals in provision of sexual and reproductive health: A scoping review

Ma. Veronica Cruz, Ma. Vinna Cruz, Maheeka Seneviwickrama

30 October 2024

New onset diabetes mellitus post COVID‐19 infection: A systematic review and meta‐analysis

Emma Cocking, Joseph Daher, Majid Alabbood

8 October 2024

The application of information and communication technologies in nutritional interventions for breast cancer: A scoping review

Xin Chen, Yutong Yi, Maaz Imam, J. J. Pionke, Lixcy Vega, Anna Arthur, Jessie Chin, Chungyi Chiu

4 October 2024

Explainable machine learning for breast cancer diagnosis from mammography and ultrasound images: A systematic review

Daraje Gurmessa, Worku Jimma

3 October 2024

## SOCIAL WELFARE

Effectiveness of artificial intelligence‐based psychotherapy in treating mental disorders

Amit Soni, Vinit Singh, Mohit Kumar

2 September 2024

## DISCONTINUED PROTOCOLS

If you are interested to continue one of the projects below, please get in touch with the Managing Editor of the relevant Coordinating Group or email info@campbellcollaboration.org.

## CRIME AND JUSTICE

https://onlinelibrary.wiley.com/doi/10.1002/CL2.85

PROTOCOL: Mandatory arrest for misdemeanor domestic violence effects on repeat offending

Barak Ariel, Lawrence W. Sherman

## EDUCATION

https://onlinelibrary.wiley.com/doi/10.1002/CL2.215

Protocol for a systematic review: Interventions to improve mathematics achievement in primary school‐aged children: A systematic review

Victoria Simms, Camilla Gilmore, Seaneen Sloan, Clare McKeaveney

https://onlinelibrary.wiley.com/doi/10.1002/cl2.1011

PROTOCOL: Protocol for a systematic review: Inter‐school collaborations for improving educational and social outcomes for children and young people: A systematic review

Paul Connolly, Jennifer Hanratty, Joanne Hughes, Christopher Chapman, Danielle Blaylock

https://onlinelibrary.wiley.com/doi/10.1002/CL2.145

PROTOCOL: Practices and program components for enhancing prosocial behavior in children and youth: A systematic review

Asha L. Spivak, Mark W. Lipsey, Dale C. Farran, Joshua R. Polanin

https://onlinelibrary.wiley.com/doi/10.1002/CL2.185

PROTOCOL: The direct and indirect effects of school‐based executive function interventions on children and adolescents' executive function, academic, social‐emotional, and behavioral outcomes: A systematic review

Saiying Steenebergen‐Hu, Paula Olszewski‐Kubilius, Eric Calvert

https://onlinelibrary.wiley.com/doi/10.1002/CL2.163

PROTOCOL: Provision of information and communications technology (ICT) for improving academic achievement and school engagement in students aged 4–18

Kristin Liabo, Laurenz Langer, Antonia Simon, Kathy‐Ann Daniel‐Gittens, Alex Elwick, Janice Tripney

https://onlinelibrary.wiley.com/doi/10.1002/CL2.210

PROTOCOL: Universal school‐based programmes for improving social and emotional outcomes in children aged 3–11 years

Paul Connolly, Sarah Miller, Jennifer Hanratty, Jennifer Roberts, Seaneen Sloan

https://onlinelibrary.wiley.com/doi/10.1002/CL2.110

PROTOCOL: Education support services for improving school engagement and academic performance of children and adolescents with a chronic health condition

Michelle A. Tollit, Susan M. Sawyer, Savithiri Ratnapalan, Tony Barnett

https://onlinelibrary.wiley.com/doi/10.1002/CL2.197

PROTOCOL: Electronic mentoring to promote positive youth outcomes for young people under 25: A systematic review

Robyn M. O'Connor, David L. DuBois, Lucy Bowes

https://onlinelibrary.wiley.com/doi/10.1002/CL2.166

PROTOCOL: Merit pay programs for improving teacher retention, teacher satisfaction, and student achievement in primary and secondary education: A systematic review

Gary Ritter, Julie Trivitt, Leesa Foreman, Corey DeAngelis, George Denny

## INTERNATIONAL DEVELOPMENT

https://onlinelibrary.wiley.com/doi/10.1002/cl2.1122

PROTOCOL: Development evaluations in India 2000–2018: A country impact evaluation map

Ashrita Saran, Eti Rajwar, Bhumika T. V., Divya S. Patil, Howard White

https://onlinelibrary.wiley.com/doi/10.1002/cl2.1186

PROTOCOL: The association between whole‐grain dietary intake and noncommunicable diseases: A systematic review and meta‐analysis

Wasim A. Iqbal, Gavin B. Stewart, Abigail Smith, Linda Errington, Chris J. Seal

https://onlinelibrary.wiley.com/doi/10.1002/CL2.84

PROTOCOL: Nutrition interventions and programs for reducing mortality and morbidity in pregnant and lactating women and women of reproductive age: A systematic review

Philippa Middleton, Caroline Crowther, Tanya Bubner, Vicki Flenady, Zulfiqar Bhutta, Tran Son Trach, Zohra Lassi

https://onlinelibrary.wiley.com/doi/10.1002/CL2.138

PROTOCOL: Improving maternal, newborn and women's reproductive health in crisis settings

Primus Che Chi, Henrik Urdal, Odidika U. J. Umeora, Johanne Sundby, Paul Spiegel, Declan Devane

https://onlinelibrary.wiley.com/doi/10.1002/CL2.183

PROTOCOL: The impacts of interventions for female economic empowerment at the community level on human development

Marcela Ibanez, Sarah Khan, Anna Minasyan, Soham Sahoo, Pooja Balasubramanian

https://onlinelibrary.wiley.com/doi/10.1002/CL2.154

PROTOCOL: Peer‐based interventions for reducing morbidity and mortality in HIV‐positive women

J. Petkovic, M. Doull, A. M. O'Connor, J. Aweya, M. Yoganathan, V. Welch, G. A. Wells, P. Tugwell

https://onlinelibrary.wiley.com/doi/10.1002/CL2.188

PROTOCOL: Community‐led total sanitation in rural areas of low‐ and middle‐income countries: A systematic review of evidence on effects and influencing factors

Josef Novotný, Jiří Hasman, Martin Lepič, Vít Bořil

https://onlinelibrary.wiley.com/doi/10.1002/CL2.140

PROTOCOL: Access to electricity for improving health, education and welfare in low‐ and middle‐income countries

Kavita Mathur, Sandy Oliver, Janice Tripney

https://onlinelibrary.wiley.com/doi/10.1002/CL2.157

PROTOCOL: Saving promotion interventions for improving saving behaviour and reducing poverty in low‐ and middle‐income countries

Janina Isabel Steinert, Ani Movsisyan, Juliane Zenker, Ute Filipiak, Yulia Shenderovich

https://onlinelibrary.wiley.com/doi/10.1002/CL2.172

PROTOCOL: The effect of interventions for women's empowerment on children's health and education

Sebastian Vollmer, Sarah Khan, Le Thi Ngoc Tu, Atika Pasha, Soham Sahoo

https://onlinelibrary.wiley.com/doi/10.1002/CL2.200

PROTOCOL: The effects of road infrastructure, and transport and logistics services interventions on women's participation in informal and formal labour markets in low‐ and middle‐income countries

Manisha Gupta, Souvik Bandyopadhyay, Meerambika Mahapatro, Shreya Jha

https://onlinelibrary.wiley.com/doi/10.1002/cl2.1135

PROTOCOL: Water, sanitation and hygiene for reducing childhood mortality in low‐ and middle‐income countries

Hugh Sharma Waddington, Sandy Cairncross

## SOCIAL WELFARE

https://onlinelibrary.wiley.com/doi/10.1002/cl2.1056

PROTOCOL: Psychosocial interventions for preventing PTSD in children exposed to war and conflict‐related violence: A systematic review

Jennifer Hanratty, Laura Neeson, Tania Bosqui, Michael Duffy, Laura Dunne, Paul Connolly

https://onlinelibrary.wiley.com/doi/10.1002/cl2.1259

PROTOCOL: Effects of virtual reality exposure therapy versus in vivo exposure in treating social anxiety disorder in adults: A systematic review and meta‐analysis

Martin Polak, Norbert Tanzer, Per Carlbring

https://onlinelibrary.wiley.com/doi/10.1002/CL2.109

PROTOCOL: The therapeutic alliance and psychotherapy outcomes for young adults aged 18 to 34

Stacy J. Green, Julia H. Littell, Karianne Hammerstrøm, Emily Tanner‐Smith, Jessica Schaffner Wilen

https://onlinelibrary.wiley.com/doi/10.1002/CL2.56

PROTOCOL: Cognitive‐behavioural therapy for parents who have physically abused their children

Mogens N. Christoffersen, Jacqueline Corcoran, Diane DePanfilis, Claire Daining

